# Serological and molecular characterization of hepatitis B virus in asymptomatic blood donors in Iran

**Published:** 2018-02

**Authors:** Mona Seyed Attaran, Seyed Masoud Hosseini, Javad Fakhari, Zohreh Sharifi

**Affiliations:** 1 Blood Transfusion Research Center, High Institute for Research and Education in Transfusion Medicine, Tehran, Iran; 2 Department of Microbiology, Faculty of Life Sciences and Technology, Shahid Beheshti University, Tehran, Iran

**Keywords:** Hepatitis B virus, Serological markers, Viral load, Iran

## Abstract

**Background and Objectives:**

Hepatitis B virus (HBV) is an enveloped DNA virus belongs to Hepadnaviridea family. HBV infection is a serious global health problem, with 2 billion people infected worldwide, and 350 million suffering from chronic HBV infection. The aim of this study was to determine the serological pattern and molecular characterization of HBV diversity in asymptomatic blood donors in Iran alongside with the mutation status of HBV in relation to blood safety.

**Materials and Methods:**

One hundred and sixty six samples from asymptomatic blood donors who were positive for hepatitis B surface antigen during 2012 to 2014 were selected. The serological and molecular markers were analyzed by screening HBsAb, HBcAb, HBeAg and HBeAb and HBV-DNA. For detection of HBV genotypes and possible mutations, HBV polymerase and pre core/core regions were sequenced.

**Results:**

In term of serologic markers of HBV, 100% of asymptomatic blood donors were HBsAg positive and 97.6%, 92.2%, 5.4% and 2.4% of them were HBcAb, HBeAb, HBeAg and HBsAb positive respectively in asymptomatic blood donors. The maximum of samples viral load was 4.41 × 10^7^ IU/ml for HBeAg positive blood donors. While the minimum and maximum of viral load in HBeAb positive samples was 1.21 × 10^2^ IU/ml and 1 × 10^6^ IU/ml respectively and the mean of viral load in HBeAb positive samples was 5.882 × 10^3^ IU/ml. About 9.7% of HBeAb positive samples had a pre core mutation that is related to stopping the synthesis of HBeAg and only genotype D was prevalent in asymptomatic blood donors.

**Conclusion:**

This study showed that from 166 samples most of them were in a chronic phase of HBV infection and just 5.4% of asymptomatic blood donors were in the acute phase or acute chronic phase of HBV infection. The major risk factor for HBV infection was a familial history of HBV.

## INTRODUCTION

Hepatitis B virus (HBV) is an enveloped double-stranded DNA virus in Hepadnaviridea family that causes acute and chronic hepatitis in humans (1). HBV causes acute and chronic liver disease. About 2 billion people worldwide have been infected with Hepatitis B and 350 million are chronic HBV carriers and 600,000 die each year from HBV- related liver disease or hepatocellular carcinoma (2). HBsAg has been identified in body fluids including semen, saliva, and serum that last is more infectious than other two routes. The two non-percutaneous routes considered to have the greatest impact are intimate especially sexual contact and perinatal transmission. Perinatal transmission occurs primarily in infants born to HBsAg carrier mothers or mothers with acute hepatitis B during the third trimester of pregnancy or during the early postpartum period. But epidemiologic evidence suggests that most infections occur approximately at the time of delivery. The chance of perinatal transmission of HBV correlates with the presence of HBeAg. About 90% of HBeAg positive mothers, but only 10–15% of HBeAb positive mothers transmit HBV infection to their children. The prevalence of chronic HBV infection varies geographically, from a high rate of more than 8% in developing countries with large population, such as South-East Asia, China, sub-Saharan Africa, and the Amazon Basin, to an intermediate rate between 2% and 7% in parts of Eastern and Southern Europe, the Middle East, Japan and parts of South America, and with a low rate of infection (less than 2%) in most developed countries, such as North America, Northern and Western Europe and Australia (3). In 1979 in Iran, the prevalence of HBsAg was reported between 2.5–7.2%. In 1980, 35% of Iranian has been exposed to the HBV that estimated about 3% of Iranian population were chronic carriers differing from 1.7% to 5% in different provinces. Prevalence of HBV infection in the general population in Iran estimated 2.14% (4).

The prevalence of HBsAg in blood donors dramatically declined from 1.23% in 2001 to 0.25% in 2010 in Iran. In Sistan & Baloochestan province HBsAg prevalence decreased from 3.29% in 2001 to 0.66% in 2010 and in Fars province, the rate of HBsAg decreased from 0.82% in 2001 to 0.12% in 2010 (5). Improvement of the people’s knowledge about HBV risk factors, national vaccination program since 1993 for all neonates and also vaccination of high risk groups, donor selection, donor screening could be the cause of this decrease (4).

The aim of this study was to determine the current status of hepatitis B virus in asymptomatic blood donors in Iran as well as studying the evolution status of HBV to meet blood safety requirements.

## MATERIALS AND METHODS

### Study population.

A 166 samples were selected randomly from asymptomatic blood donors collected form all provinces for the period of 2012–2014. They were positive for HBsAg by ELISA method and were confirmed as HBV-infected blood donors. All blood donors were recalled and interviewed for the collection of data including blood donation number, date of birth, sex, having risk factors (dental work, tattooing, intravenous drug injections, having a family history of HBV infection, blood transfusion).

All sera were stored at the Iranian Blood Transfusion Organization (IBTO) at −70°C for further use. All samples were analyzed for the presence of Hepatitis C virus (HCV), Hepatitis D virus (HDV), Human immunodeficiency virus (HIV) and HBV serological markers using ELISA kits, and also viral load, HBV genotype and identifying mutation in core regions of the virus genome.

### ELISA.

HBsAg positive sera were tested for HB-cAb (Cut-off = (NC + PC) /5; OD = 450 nm; positivity of >1.1), HBeAg (Cut-off = (NC + 0.1); OD = 450 nm; positivity of >1/1), HBeAb (Cut-off = (NC + PC)/3; OD = 450 nm; positivity of >1.1) and HBsAb, (DIA.PRO, Italy) according to the manufacturer’s instructions for all tests.

### Viral nucleic acid extraction.

Hepatitis B virus DNA was extracted from 200 μl serum using the High Pure Viral Nucleic Acid kit (Roche, Mann-heim, Germany) following the manufacturer’s guidelines and then extracted DNA was suspended in 50 μl of elution buffer.

### HBV-DNA and viral load.

PCR mix was prepared in a total volume of 50 μl for samples containing 25 μl Master Mix (in-house, IBTO kit), 1 μl of each (10 μM) primers for surface region synthesized at the Metabion International AG Company (Martinsried, Germany) (Forward: 5′- GCATGGARACCACCGTGAAC -3′ and Reverse: 5′- CGATACAGAGCWGAGGCGGT -3′) and 5 μl DNA template. The amplification was carried out under the following conditions: 3 minutes initial step at 95°C, followed by 40 cycles of amplification including denaturation at 95°C for 1 minute, annealing for 1 minute at 55°C, and extension at 72°C for 1 minute, one cycle including final extension at 72°C for 5 minutes and final hold at 4°C. For prevention of false positive and negative results, we used plasma negative and positive samples in each run. The lower limit of detection of the PCR was 20 IU/ml (6).

Detection of HBV viral load using Light Cycler Roche. 2 was performed. Real-Time PCR was done by using Real ART HBV LC PCR kit (Artus GmbH, Hamburg, and Germany) according to the manufacturer’s instructions (7).

### Sequencing.

All HBsAg and HBeAb positive sera with viral load > 2000 were amplified and sequenced for analyzing mutation of pre-core and core regions of virus genome with 1 μl of each (10 μM) primers for core region (Forward: 5′-GCATGGARACCACCGTGAAC-3′ and Reverse: 5′- CGATACAGAGCWGAGGCGGT -3′) and 5 μl DNA template. The amplification was carried out under the following conditions: 3 minutes initial step at 95°C, followed by 35 cycles of amplification including denaturation at 95°C for 1 minute, annealing for 45 seconds at 60°C, and extension at 72°C, one cycle including final extension at 72°C for 10 minutes and final hold at 4°C (8).

HBV genotypes were determined by direct sequencing of the HBV polymerase gene. Phylogenetic trees were constructed by the neighbor-joining (NJ) method with 1000 bootstrap replicates.

## RESULTS

All 166 sera including 95% male and 5% female with the average age of 38±10 years old, ranging from 18–60 years were positive for HBsAg. Serum ALT and AST levels were normal, and ranged between 10–28 IU/L (16.7±6.4, mean_SD) and 6–29 IU/L (mean 17.4±5.6), respectively. About 71% of the blood donors were first-time donors, 27% and 2% of them lapsed and regular donors, respectively. The frequency of Hepatitis B virus infection increased with age. Also, 27% of infected blood donors had risk-factor dental works, 32% mentioned familial history of HBV, 17%, 9%, 1.2% and 0.6% had tattooing, history of hospitalization, phlebotomy and intravenous drug injections, respectively and 13.2% had not encountered with any risk-factor (Table 1).

**Table 1.** Association between HBs antigen positivity and risk factors in HBV infected asymptomatic blood donors

**Risk factor**	**HBsAg positivity**		**p value**

	**Number**	**(%)**	
Dental practice	46	27	>0.05
Having HBV positive patients in the family	54	32	<0.00
Tattooing	29	17	>0.05
History of hospitalization	15	9	>0.07
Phlebotomy	2	1.2	>0.05
Intravenous drug injections	1	0.6	>0.05

In terms of serologic markers of HBV, 100% of asymptomatic blood donors were HBsAg positive and 97.6%, 92.2%, 5.4% and 2.4% of them were HB-cAb, HBeAb, HBeAg and HBsAb positive, respectively (Table 2).

**Table 2.** The results of serological markers of HBV in HBV-infected asymptomatic blood donors

	**anti-HBc**		**anti-HBs**		**HBeAg**		**anti-HBe**		**anti-DeltaAg**		**anti-HCV**		**anti-HIV**	

	**No.**	**%**	**No.**	**%**	**No.**	**%**	**No.**	**%**	**No.**	**%**	**No**	**%**	**No.**	**%**
Positive	162	97.6	4	2.4	9	5.4	153	92.2	0		0		0	
Negative	4	2.4	162	97.6	157	94.6	13	7.8	166	100	166	100	166	100

HBV viral load were performed on all 166 samples; the maximum viral load was 4.41 × 10^7^ IU/ml for HBeAg positive blood donors. While the maximum and minimum viral load in HBeAb positive samples was 1× 10^6^ IU/ml and 1.21 × 10^2^ IU/ml, respectively and the mean for viral load in HBeAb positive samples was 5.882 × 10^3^ IU/ml.

HBV genotyping were determined by direct sequencing of the HBV polymerase gene and only genotype D was prevalent in asymptomatic blood donors; also none of them had mutations associated with lamivudine resistance.

Direct sequencing of the HBV core gene showed that about 9.7% of HBeAb positive samples had the precore mutation that is related to stopping the synthesis of HBeAg. The predominant mutation of the precore region was a G to A substitution change at nucleotide 1896, which creates a premature stop codon at codon 28. Blood donors that had Mutations in precore region of HBV were with the high viral load.

About 52% of samples had two mutations of A1762T and G1764A that is the most prevalent mutations in the basal core promoter (BCP).

## DISCUSSION

Hepatitis B infection is the 10^th^ leading cause of death by chronic hepatitis, cirrhosis, and hepatocellular carcinoma (HCC) in the world. The prevalence of HBV infection varied widely, with rates ranging from 0.1% to 20% in different parts of the world. Overall, 45% of the world population lives in high prevalence regions (9).

Three phases of chronic HBV infection are now widely accepted. The immune tolerant phase, the immune active phase, and the inactive hepatitis B phase. In the immune tolerant phase levels of HBV DNA are > 20,000 IU/ml with HBeAg positive and normal ALT levels. The immune active phase also sometimes referred to as the chronic hepatitis B phase or the immune clearance phase is characterized by elevated ALT levels and an elevated HBV DNA level above at least 2000 IU/ml. The inactive hepatitis B phase is characterized by the absence of HBeAg and the presence of HBeAb normal ALT levels, HBV DNA < 2000 IU/ml and improvement in liver fibrosis and inflammation over time (10).

In this study, 166 samples were collected and HBV serology markers were tested for all sera and then confirmed by molecular assays. From 166 samples, in 9 (5.4%) and in 100% of samples HBeAg and HBV-DNA were detected, respectively, indicated all of them were in the high transmissible phase of the disease. In 5.4% of HBeAg positive samples, HBV-DNA levels were ≥ 2×10^7^ IU/ml and they were in the acute phase or acute chronic phase of HBV infection.

A 153 (92.2%) samples were HBeAb positive and PCR was positive for 107 (70%) of them. Previous studies showed the presence of HBV DNA in 83–100% of HBeAg positive/HBeAb negative carriers and in 26–64% of HBeAg negative/HBeAb positive cases (11). In this study, out of 107 samples that for HBV viral load were positive; 64.7% of them had HBVDNA levels< 2000 IU/ml and they were in chronic hepatitis B immune control phase or inactive Hepatitis B levels; also HBV-DNA levels in about 35.3% of HBeAb positive samples was > 2000 IU/ml and they were in HBeAg negative chronic hepatitis B phase or the immune active phase that only in 9.7% of HBe-Ab positive samples, mutation of pre core and core regions was detected. Therefore, they were in HBeAg negative chronic hepatitis B phase presumably due to mutation of pre core and core region of virus genome that causes stop or reduces HBeAg production. This mutation is common in Asia and the Mediterranean (11) and major in genotype D of HBV which is prevalent in Iran (12). In a study, 55.5% of HBsAg and HBeAb positive blood donors had an active virus infection that was due to mutation of pre core and core region of virus genome that causes stop or reduces HBeAg production (13).

High prevalence of HBeAb accompanied by HBsAg and HBcAb in current study indicated that most of blood donors had chronic hepatitis. Also, the prevalence of HBeAg/Ab negative or positive samples was 2 (1.2%). Studies indicate some patients may show other serological cases. Low antigen titer or formation of antigen-antibody complex cause HBeAg/Ab negative abnormal cases that antigen not detected with routine methods (11). In a study in England from 355 HBV-infected blood donors, 97% had chronic infection and 3% of them had an acute infection. About 7% of infected blood donors were HBeAg positive and in 91% of them, HBeAb were detected. Also, about 0.28% of samples were positive for both HBeAg and HBeAb and 1.69% of them were negative for both markers (14). But in our study, the prevalence of HBeAg was lower and the prevalence of HBeAb was higher than the study in England that is related to place of residence of blood donors (14). On the other hand, HBV genotype considered as a critical factor in the prevalence of HBeAb. In the report on HBV infected blood donors in France, 6.3% and 93.1% of HBeAg and HBeAb positive had genotype D respectively (15).

In 2.4% of samples with a familial history of HBV were HBcAb negative and HBeAg positive that can be due to immunologic tolerance in the uterus; in some infants who were born from HBeAg positive carrier mothers, HBcAb has been found to be negative (16). Additionally, in this study 2.4% of the total sample were HBsAb positive that in 50% of them HBV-DNA was detected that can be due to the amino acid mutations in the S gene region, especially in the a-determinant, and another reason may be due to another subtype of HBsAg that is not incapable of producing neutralizing HBsAb against another subtype of antigen (17). On the other hand, in the case report from Australian Red Cross Blood Service, two cases of asymptomatic HBV infection in vaccinated blood donors with protective HBsAb level was indicated that acute infection with HBV-DNA in the presence of HBsAb is demonstrated (18).

As previously reported, the most prevalent genotype in an asymptomatic blood donors was D (19). Genotype D is commonly found in the Mediterranean area and the rate of chronicity of acute genotype D infection is higher than other genotype HBV infection. In other study done on HBV genotypic in carriers in Mexico, demonstrated that diversity was more in asymptomatic carriers than symptomatic carriers (20).

In conclusion, this study showed that most blood donors were in a chronic phase of HBV infection and just 5.4% of asymptomatic blood donors had an acute phase or acute chronic phase of HBV infection. The major risk factor for HBV infection was a familial history of HBV.
